# The Effects of Honeysuckle (*Lonicera caerulea* L.) Berry Iridoid-Anthocyanin Extract on the Intestinal and Muscle Histopathology in Mice during Experimental Trichinellosis

**DOI:** 10.3390/molecules28207067

**Published:** 2023-10-13

**Authors:** Jolanta Piekarska, Jan P. Madej, Michał Gorczykowski, Marianna Szczypka

**Affiliations:** 1 Division of Parasitology, Department of Internal Medicine and Clinic of Horses, Dogs and Cats, Faculty of Veterinary Medicine, Wroclaw University of Environmental and Life Sciences, Norwida 31, 50-375 Wroclaw, Poland; michal.gorczykowski@upwr.edu.pl; 2 Department of Immunology, Pathophysiology and Veterinary Preventive Medicine, Faculty of Veterinary Medicine, Wroclaw University of Environmental and Life Sciences, Norwida 31, 50-375 Wroclaw, Poland; jan.madej@upwr.edu.pl; 3 Department of Pharmacology and Toxicology, Faculty of Veterinary Medicine, Wroclaw University of Environmental and Life Sciences, Norwida 31, 50-375 Wroclaw, Poland; marianna.szczypka@upwr.edu.pl

**Keywords:** *Lonicera caerulea* L., *T. spiralis*, intestine, muscle, morphology

## Abstract

The aim of the study was to determine the effect of iridoid-anthocyanin extract from honeysuckle (*Lonicera caerulea* L.) (LC) berries on histopathological changes in the intestines and muscles during experimental trichinellosis in mice. The LC extract was administered to uninfected mice (LC group) and *Trichinella-spiralis*-infected mice (T+LC) orally at a dose of 2 g/kg bw, six times at 24 h intervals, from day 3 prior to infection to day 3 post-infection (dpi). Jejunum samples were collected on 5, 7, 14, and 21 dpi, and their histological assessment involved the villus height to crypt depth ratio (VH/CD), goblet cell (GC) number, and morphological changes. In the *T. spiralis*-infected muscles, the extent of inflammatory infiltration on the 14th and 21st dpi was assessed. LC in the infected mice restored the VH/CD ratio to control values on 14 dpi. A beneficial effect of the LC extract on the villus height was also observed 14 dpi in the LC and T+LC groups. No differences in the extent of inflammatory infiltration in the muscles between the T+LC and T groups were observed. In conclusion, the iridoid-anthocyanin extract from honeysuckle berry contributed to alleviating the symptoms of the intestinal phase of *T. spiralis* infection.

## 1. Introduction

Inspired by observations and experiences of folk medicine, numerous scientific studies have been conducted on the mechanisms of the healing, protective, and stimulating properties of the substances contained in honeysuckle (*Lonicera caerulea* L.) (LC) berries. The beneficial effects of LC fruits were found to be conferred by biologically active substances such as vitamins E, B2, B1, and C, folic acid, anthocyanins, iridoids (e.g., loganic acid), or flavonoids [1,2]. As modulators of inflammatory processes, these compounds exhibit, among others, anti-inflammatory, antimicrobial, anticancer, and antioxidant properties [1,2]. The antiparasitic effect of specific biologically active substances has also been demonstrated. Flavonoid compounds widely present in plants exert anti-amebic activity [3]. Iridoids and plant flavonols (e.g., quercetin) have demonstrated medicinal activity in different clinical trials, such as those against *Leishmania*, *Trypanosoma*, and *Plasmodium* [4,5,6].

Trichinellosis is a widespread parasitic disease caused by eating meat products contaminated with the first-stage larvae of nematodes belonging to *Trichinella genus*. *Trichinella spiralis* is an unusual parasite, whose invasion occurs in two phases, the intestinal and the muscular one, and whose life cycle is completed in a single host. The parasite is one of the most dangerous human parasites responsible for a severe and sometimes fatal course of the disease. Numerous studies have been carried out around the world in search for plant-based therapeutics [7,8,9]. To combat trichinellosis, it is important to establish whether active substances of plant origin affect both the host immune system and the parasite.

We previously studied the effects of other plant extracts containing the above-mentioned active substances on the course of experimentally induced trichinellosis in mice [10,11]. Aqueous extract from *Scutellaria baicalensis* Georgi roots, containing mainly flavonoids as active compounds, did not significantly change the number of intestinal parasites or the number of muscle larvae (only a downward trend was observed) [11]. Another study showed that iridoid-anthocyanin extract of *Cornus mas* L. significantly reduced the intestinal parasite load on the 5th day after infection. However, it did not affect the number of muscle larvae [10].

This study is a continuation of our previous research, in which we demonstrated that natural iridoid-anthocyanin extract from LC modulates the murine cellular immune response during the intestinal phase of *T. spiralis* infection, but shows no antiparasitic activity [12]. There, we reported that aqueous LC extract, administered to mice in the course of experimentally induced trichinellosis, affected the proliferative activity of splenocytes, altered the percentage and absolute count of B (CD19^+^) and T (CD3^+^, CD8^+^) cells in the peripheral blood, and modified selected hematological parameters. Although the immunotropic activity of LC did not expressly change the intensity of the *T. spiralis* infection, its immunomodulatory properties could be used to mitigate the intestinal inflammation associated with trichinellosis [12].

During the course of *T. spiralis* infection, it is also very important to determine whether there is a protective or beneficial effect of the active substances against the development of acute pathology in the intestinal and muscular phases, which may be life-threatening. Therefore, the aim of this study was to assess the effects of in vivo administered iridoid-anthocyanin extract from honeysuckle berry on the histopathological changes in the intestine and muscles during experimental trichinellosis in mice.

## 2. Results

### 2.1. Morphological Analysis (HE)

#### 2.1.1. Effect of LC on Morphology of the Jejunum

**Figure 1:**
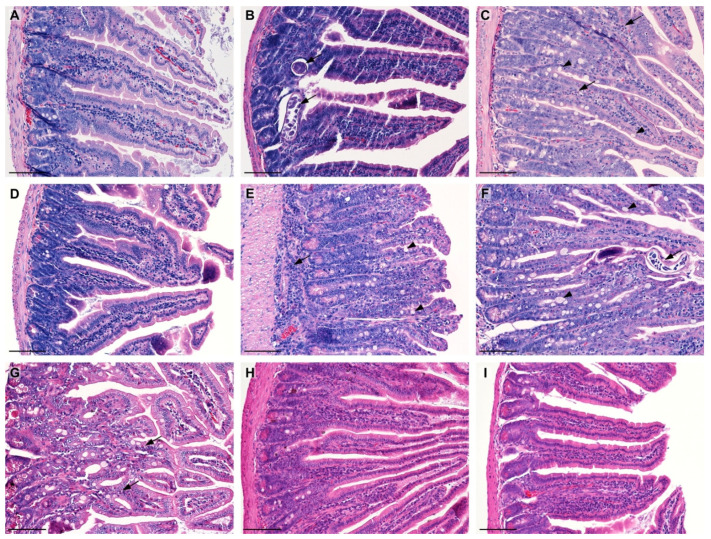
Selected histopathological views of the murine jejunum. (**A**)—normal structure of the jejunum with long intestinal villi and shallow intestinal glands (crypts) 5 dpi (C group); (**B**)—adult *T. spiralis* forms visible 5 dpi (T group); (**C**)—5 dpi T+LC—focal eosinophilic infiltrates in the villi (arrow), goblet cell hyperplasia (arrowhead), and a marked increase in the depth of the glands; (**D**)—7 dpi, control group with normal structure of the jejunum; (**E**)—7 dpi, T group, visible infiltration of the inflammatory cells in the lamina propria of the mucosa with local eosinophilic infiltrates (arrow), goblet cell hyperplasia (arrowhead), strong shortening of the villi, and a marked increase in the depth of the glands; (**F**)—7 dpi, T+LC group, adult worms (arrow) and goblet cell hyperplasia (arrowhead) visible in the mucosa, slight shortening of the villi and a marked increase in the depth of the glands; (**G**)—14 dpi, T group, eosinophilic infiltration in the mucosa; and (**H**,**I**)—21 dpi, restoration of the normal length of the intestinal villi and the depth of the crypt in T (**H**) and T+LC (**I**) groups. H&E staining. Scale bar = 100 µm.

In the control group (C), the jejunum mucosa was normal at all time points, with long intestinal villi and shallow intestinal glands (crypts) (Figure 1A). Single infiltrative cells within the connective tissue of the intestinal villi were observed.

In the LC group 5 dpi, the dilatation of some intestinal villi, moderate vasodilation (capillaries filled with erythrocytes), and a slight increase in the depth of the intestinal glands were observed. In the group infected with the parasites (T), trichinella adult forms were present under the epithelium in the wall of the intestinal villi and at the level of the intestinal glands (Figure 1B). Moreover, moderate blood vessel hyperemia, widening of the intestinal villi, and a marked increase in the depth of the glands were noted. Goblet cell hyperplasia was present. Prominent histiocytic and lymphocytic infiltration was observed in the connective tissue of the intestinal villi, while in the lamina propria of the mucosa, it was mixed with focal eosinophilic infiltrates. In the T+LC group, adult trichinella forms were also present in the wall of the intestinal villi and at the level of the intestinal glands. Histopathological examination revealed goblet cell hyperplasia, widening of blood vessels, and prominent histiocytic and lymphocytic infiltration in the connective tissue of the intestinal villi (Figure 1C). Focal eosinophilic infiltrates were also noted in the villi. Widening of the intestinal villi with a marked increase in the depth of the glands was demonstrated.

In the LC group 7 dpi, the capillaries of the jejunum showed slight hyperemia. The length of the villi and the depth of the intestinal glands did not differ from the control (Figure 1D). In the T group, marked goblet cell hyperplasia, mild vasodilation, mild histio-lymphocytic infiltration in the apical parts of the intestinal villi, and moderate infiltration of inflammatory cells in the lamina propria of the mucosa with local eosinophil infiltrates were observed. Severe shortening and widening of the intestinal villi, as well as a considerable increase in the depth of the glands were present (Figure 1E) In the T+LC group, noticeable goblet cell hyperplasia, mild vasodilation, mild histio-lymphocytic infiltration in the apical parts of the intestinal villi, and moderate infiltration of the inflammatory cells with local eosinophilic infiltrates in the lamina propria of the mucosa were found. In the wall of the intestinal villi, the presence of single trichinella worms was noted (Figure 1F). Slight shortening and widening of the intestinal villi and a marked increase in the depth of the glands were observed.

In the LC-receiving group 14 dpi, the jejunum showed a mild focal dilatation of the blood vessels. In addition, the intestinal villi were longer than in the control group. In the T group, the local dilatation of blood vessels, mild goblet cell hyperplasia, and intense scattered eosinophilic infiltrates in the lamina propria of the mucosa between the glands were observed (Figure 1G). The intestinal villi reached their normal (similar to control) length, which indicated their regeneration in relation to day 7 post-infection. In the T+LC group, local eosinophilic infiltrates were found in the lamina propria of the mucosa. In addition, an increase in the depth of the glands was observed, but not as strong as that in the T group.

At 21 dpi, the jejunum morphology in the LC group did not differ from that in the control. In the T group, moderate hyperemia and focal eosinophilic infiltrates in the lamina propria of the mucosa were found. These changes were accompanied by widening of the intestinal villi and a slight increase in the depth of the glands. In the T+LC group, dilated blood vessels and numerous eosinophilic infiltrates appeared in the lamina propria of the mucosa (Figure 1H,I).

#### 2.1.2. Morphometry

**Figure 2:**
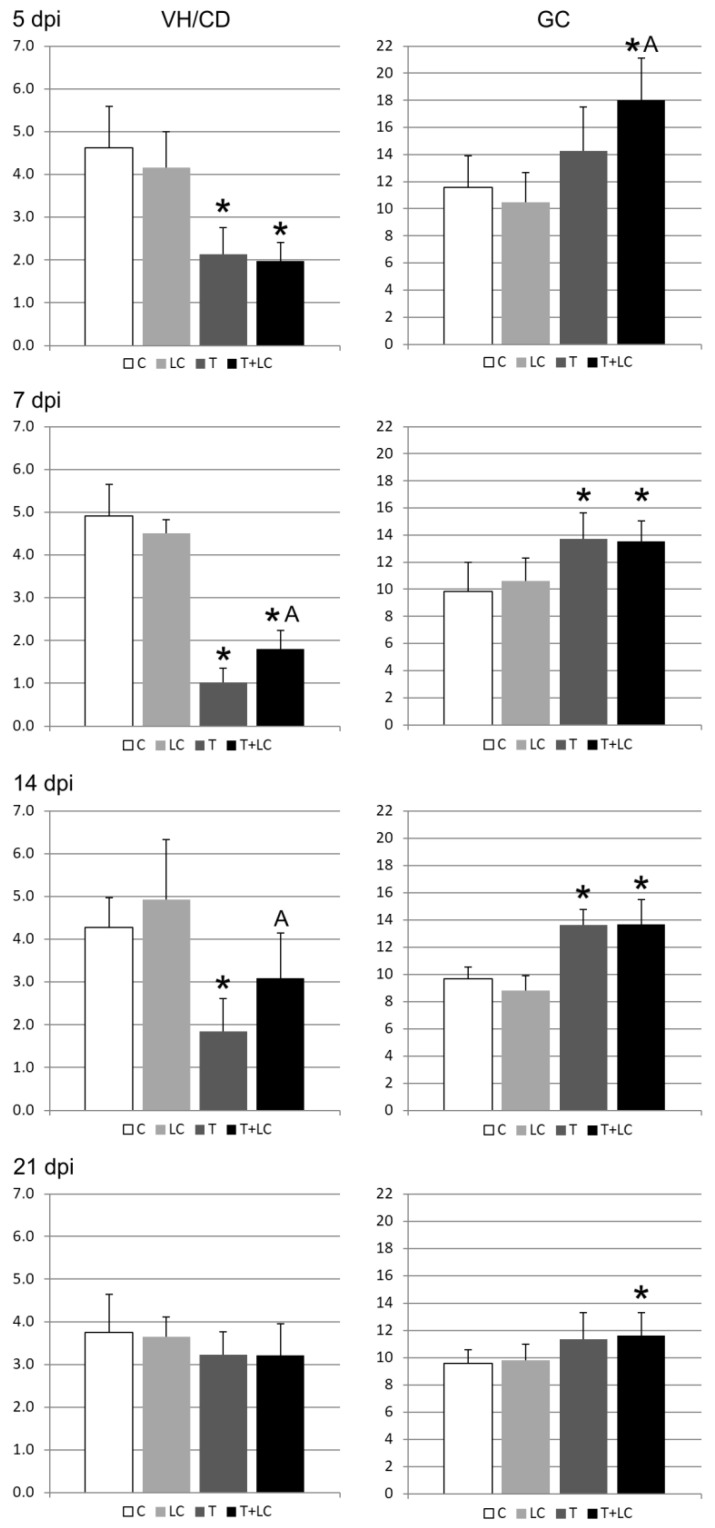
The villus height to crypt depth ratio (VH/CD) and goblet cell (GC) number (in the epithelium of one villus and one intestine gland) 5, 7, 14, and 21 dpi after *T. spiralis* and honeysuckle berry (*Lonicera caerulea*) extract administration. C—control, LC—*L. caerulae*, T—*T. spiralis*, and T+LC—*T. spiralis + L. caerulea.* Significant differences (*p* < 0.05) in comparison with the control group (*) and the T group (A).

At 5 dpi in the T and T+LC groups, the VH/CD ratio significantly (*p* < 0.05) decreased in comparison to the control groups. This change resulted from a significant increase in the crypt depth. At the same time, the number of goblet cells increased (*p* < 0.05) in the T+LC group in comparison to the control and T groups. A decrease in the villus height was observed in the T group in comparison to all other groups. (Table 1, Figure 2).

At 7 dpi, a significant decrease in the VH/CD ratio was observed in the T and T+LC groups in comparison to the control. However, the value of this ratio in the T group was significantly lower than that in the T+LC group. In the T group, it was due to the increase in CD with a simultaneous decrease in VH, while, in the T+LC group, only an increase in CD was noted. This was accompanied by an increase in the number of GCs in the T and T+LC groups. (Table 1, Figure 2).

At 14 dpi, the VH/CD ratio in the T group was significantly lower than that in the control and T+LC groups. A beneficial effect of LC on the villus height was observed in the LC and T+LC groups, where the values of this parameter were even higher than that in the control. The crypt depth was greater in the T and T+LC groups, with a much stronger effect in the T group. The goblet cell number was significantly higher in the T and T+LC groups than in the control group. (Table 1, Figure 2).

At 21 dpi, the VH/CD ratio, VH, and CD were similar in all the groups. Only the number of GCs was significantly higher in the T+LC group than in the control group. (Table 1, Figure 2).

#### 2.1.3. Effect of LC on the Morphology of the Masseter Muscles

**Figure 3:**
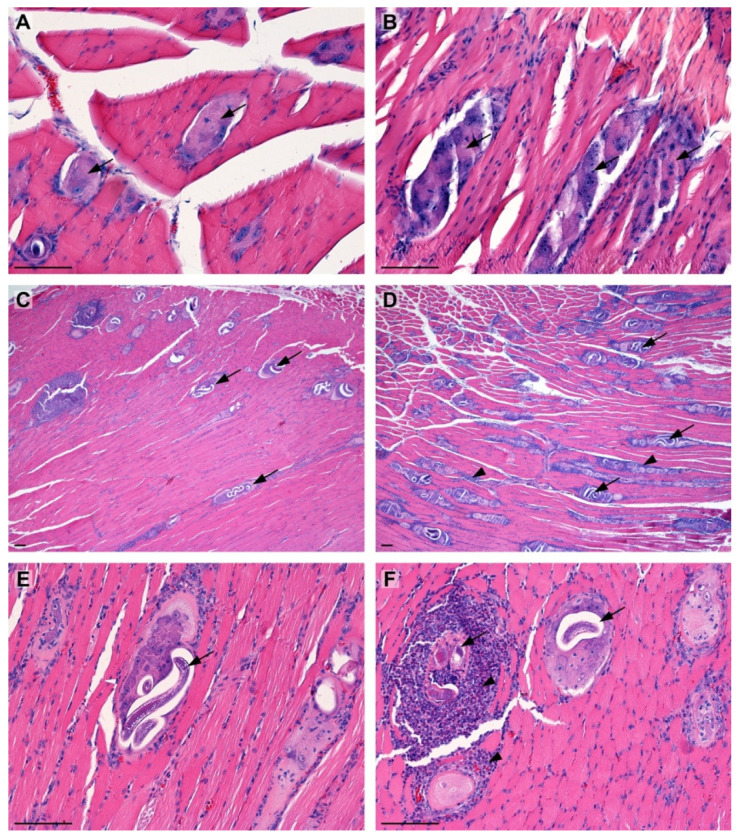
Histopathology of the mouse masseter muscle after *T. spiralis* invasion. Larvae (arrows) clearly visible in the muscle 14 dpi in the T (**A**) and T+LC group (**B**). Massive invasion of the larvae (arrows) 21 dpi in the T (**C**) and T+LC group (**D**). Characteristic coil shape of *T. spiralis* larvae 21 dpi in the specimen from the T group (**E**). (**F**)—intense infiltration of granulocytes with predominance of eosinophils (arrowhead) around the larvae (arrows). H&E staining. Scale bar = 100µm.

At 14 dpi, the masseter muscles of the animals from the T group contained numerous non-encapsulated larvae of *T. spiralis*. Similarly, in the T+LC group, numerous, non-encapsulated larvae of the parasite with a focal weak inflammatory infiltrate around the larvae were found (Figure 3A,B).

At 21 dpi, numerous encapsulated larvae at various stages of development were found in the striated muscles of the T group animals, along with the accompanying infiltration of granulocytes with a predominance of eosinophils. Similarly, in the T+LC group, the muscles contained a large number of encapsulated *T. spiralis* larvae at various stages of development. Around the larvae, an intense infiltration of granulocytes, mainly eosinophils, was also observed (Figure 3D,E).

## 3. Discussion

Trichinellosis is still a topical, cosmopolitan parasitic zoonosis with direct relevance to human and animal health, which also presents an economic problem in porcine animal production and food safety [13].

The pathogenesis of the disease is associated with the unique life cycle of *Trichinella* nematodes, and it involves numerous structural, cellular, and physiological changes and an acute inflammatory response in the small intestine and striated muscle of the host [14]. At the first stage of the invasion, the larvae of *T. spiralis* invade the epithelium of the small intestine, reach their adult stage there, and produce live-born larvae. These newborn larvae (NBL) penetrate the submucosa of the small intestine and migrate through the circulatory system to the striated muscle cells. In the muscle phase, NBL grow and mature to the infectious L1 stage, completing the life cycle [15,16]. In each phase of the life cycle, both the adults and larvae of *T. spiralis* produce different antigenic components (excretory/secretory antigens), which directly influence the host immune response [17].

Intestinal invasion is a very important step, as it determines the intensity of the muscle phase and the course and consequences of trichinellosis for the host [14]. The effective pharmacotherapy of trichinellosis with antiparasitic and steroidal anti-inflammatory drugs is limited by side effects and resistance, and so more safe and effective drugs, particularly plant-based ones, are needed [7]. Studies on parasites have largely focused on plant components that are important or essential for the parasite’s biology. Various active plant compounds have been identified and characterized as immunomodulators that successfully modulate the host response pathway during *T. spiralis* infection [18,19,20]. Some plants with potential prophylactic and therapeutic activity against *T. spiralis* in its intestinal and muscular phases have also been described. An extract of *Annona muricata* (Graviola) administered to mice infected with *T. spiralis* provided therapeutic results comparable with albendazole (ABZ), and could serve as an adjuvant to anti-trichinellosis therapy [21]. Similar results were reported for *Punica granatum* peel extract, which may be used as a new agent in trichinellosis treatment, particularly when combined with ABZ [22].

In our previous work, we found that iridoid-anthocyanin extract from honeysuckle berry affected the proliferative activity of splenocytes and altered the percentage and absolute count of B (CD19^+^) and T (CD3^+^, CD8^+^) cells in the peripheral blood of mice infected with *T. spiralis* [12]. Although the LC extract affected the dynamics of the expulsion of adult *Trichinella* from the intestines, it did not significantly change the number of adult parasites in the intestines or the number of larvae in the muscles [12].

Our present study indicated that, at 5 dpi, the treatment with aqueous LC extract significantly increased the number of intestinal goblet cells in the course of trichinellosis in mice. Goblet cells and the mucus they secrete play significant roles in the protection of the intestinal epithelium from intestinal pathogens [23,24]. An increased number of goblet cells as well as qualitative changes in the mucus composition accompany the invasion of multiple nematode species, including *T. spiralis* [25,26,27]. Intestinal goblet cells are crucial for promoting intestinal defense against parasites, viruses, and bacteria thanks to secreting trefoil factor peptides (TFF), mucins, Fc-γ binding protein (Fcgbp), or resistin-like molecule β (RELMβ), which help to actively detect and respond to infections or improve the regeneration of the mucosa [24,28,29].

Host immune response factors, particularly Th2 cells, play a major role in the development of goblet cells during intestinal trichinellosis [30]. Increased activity of the mucus-producing cells in the LC-treated group of *T. spiralis*-infected mice therefore confirmed the beneficial influence of the extract on the host immune system.

In our study, the LC extract influenced the typical symptoms accompanying the infection of intestinal nematodes, such as the shortening and deformation of the intestinal villi and hypertrophy of the crypts. The administration of the LC extract to the mice infected with *T. spiralis* had a beneficial effect on the VH/CD ratio by increasing the villus height and decreasing the crypt depth at 7 and 14 dpi. The mechanism of LC action may result from its specific composition of active substances and their biological properties, including an influence on lymphocyte activity and cytokine production. The important anthocyanin of LC, cyanidin-3-*O*-glucoside, downregulated Th2 cytokine production (IL-4 and IL-13), but it did not affect the synthesis of Th1 cytokine (IFN-γ, IL-12, and IL-2) [31,32]. Such a mechanism of action of major cyanidin-3-*O*-glucoside from LC extract could explain the fact that the extract, despite affecting the mechanisms of worm expulsion from the intestine (e.g., goblet cells and VH/CD ratio), did not reduce the number of parasites in the intestine.

During the early muscle phase of *T. spiralis* infection, numerous non-encapsulated and encapsulated larvae were observed at 14 and 21 dpi, respectively. Inflammatory cell infiltration noted in the muscles indicated that the host immune system responded to the presence of *Trichinella* larvae. However, there was no significant difference between the T+LC and T groups in this respect.

In summary, the administration of the LC extract to the animals infected with *T. spiralis* alleviated the local histopathological changes caused by the parasite in the small intestine, and manifested mainly in intensive shortening of the villi and deepening of the intestinal crypts. Such results and a significant increase in the number of intestinal goblet cells support a modulatory role of the LC extract in the course of trichinellosis in mice.

Considering all the effects mentioned above, we suggest that iridoid-anthocyanin extract from honeysuckle berry contributes to alleviating the symptoms of the intestinal phase of *T. spiralis* infection.

## 4. Materials and Methods

### 4.1. Preparation of the LC Extract

A mix of honeysuckle (*Lonicera caerulea* L. var. *kamtschatica* Sevast.) berries of the cultivars “Zojka”, “Wojtek”, and “Jolanta” was used in this study. The berries were collected from plantations near Skierniewice (51°57′00.4″ N 20°10′56.0″ E), Poland.

Raw LC fruits were extracted as described previously [12].

The content of iridoids was expressed as loganic acid, of anthocyanins as cyanidin 3-*O*-glucoside, of flavonols as quercetin 3-*O*-glucoside, and of phenolic acids as 5-*O*-caffeoylquinic (chlorogenic) acid equivalents (mg per 1 g of dry weight [dw]). The methods of identification and detailed contents of the main compounds of the honeysuckle berry extracts were published previously [12].

### 4.2. Animals and Treatment

The study was carried out in CFW mice (*n* = 96), males and females aged 8–10 weeks, weighing approximately 25–30 g, derived from the Centre for Genetic Engineering at the Faculty of Veterinary Medicine, Wroclaw University of Environmental and Life Sciences (No. in the register of breeders/suppliers: 021). The animals were housed in an air-conditioned room (23 ± 2 °C), with a 12 h light/12 h dark cycle, and had unlimited access to food and tap water.

The mice were randomly split into four groups of 24 individuals each: C (uninfected mice—control), LC (uninfected mice receiving LC), T (mice infected with *T. spiralis* larvae), and T+LC (mice infected with *T. spiralis* larvae and receiving LC). The mice from the T and T+LC groups were infected orally with 200 *T. spiralis* (T1, ISS1820, Poland) larvae. The infective larvae were recovered from the muscle tissue of mice infected two to three months before via digestion with a 1% pepsin/HCl solution for 1 h at 37 °C. The iridoid-anthocyanin extract from the *Lonicera caerulea* L. fruit was administered to the mice at a dose of 2 g/kg bw six times at 24 h intervals (from day 3 prior to the infection to day 3 post-infection with *T. spiralis*). The preparation was dissolved in 0.2 mL of distilled water and administered orally via a stomach tube to 24 uninfected mice and 24 mice infected with *T. spiralis*. The dose of the preparation was based on the study of Kim et al. [33].

The study protocol was approved by the Local Ethical Committee for animal experiments in Wrocław, Poland (Resolution No. 107/2018/P2).

All efforts were made during the experiments to minimize animal suffering. The European Convention for the Protection of Vertebrate Animals used for Experimental and Other Scientific Purposes, as well as national and institutional guidelines for the care and use of laboratory animals, were followed.

### 4.3. Collection of Tissue Samples and Histological Analyses

At 5, 7, 14, and 21 dpi, the mice were anesthetized by inhalation with 2–3% isoflurane (Forane, Aesica Queenborough Limited, Queenborough, UK) and sacrificed. The jejunum sections were taken from each mouse. Additionally, at 14 and 21 dpi, the masseter muscles were collected. The samples were fixed in 10% buffered formalin, embedded in paraffin blocks, and cut into 5 µm thick sections using a microtome, according to a standard histological procedure. The tissue sections after dewaxing in xylene and rehydration in alcohol series were stained using hematoxylin-eosin (HE).

#### Morphometry

Histometric measurements were made on microphotographs taken with a Nikon Eclipse 80i light microscope (Nikon, Melville, NY, USA) with a video camera, using NIS-Elements AR 2.30 software (Nikon, Melville, NY, USA). In each animal, one section that represented the best view of the villi and crypts was selected for analysis. The height of the intestinal villi (VH) was measured from the base of the villus, i.e., where the intestinal crypt turns into the villus, to its apex, while the depth of the crypt (gland) (CD) was measured from this point to the bottom of the gland. The number of goblet cells was counted in the epithelium of one intestinal villus together with the one adjacent intestinal gland.

### 4.4. Statistical Analysis

Quantitative data were subjected to statistical analysis using the Statistica 13.1 software (StatSoft Polska Sp. z o.o., Kraków, Poland). The significance of differences was appraised using a one-way analysis of variance (ANOVA) and post hoc Tukey’s test for data showing a normal distribution or the Kruskal–Wallis ANOVA and multiple comparisons of mean ranks for data that were not normally distributed. A value of *p* < 0.05 was considered as significant.

## Figures and Tables

**Table 1:** Villus height and crypt depth in the jejunum of the control ©, *Lonicera caerulea* (LC), *Trichinella spiralis* (T), and *T. spiralis + L. caerulea* (T+LC)-treated groups (values are mean ± standard deviation). The values in the same line with no common superscript differ significantly (*p* < 0.05).

Variable	C	LC	T	T+LC
Villus height (µm)				
5 dpi	492 ± 46 ^ab^	517 ± 69 ^a^	450 ± 75 ^b^	463 ± 49 ^ab^
7 dpi	471 ± 74 ^a^	460 ± 52 ^ab^	253 ± 74 ^c^	402 ± 59 ^ad^
14 dpi	427 ± 72 ^a^	524 ± 44 ^b^	458 ± 88 ^a^	540 ± 77 ^b^
21 dpi	395 ± 86 ^a^	423 ± 75 ^a^	419 ± 77 ^a^	440 ± 94 ^a^
**Crypt depth (µm)**				
5 dpi	110 ± 21 ^a^	129 ± 28 ^a^	217 ± 33 ^b^	240 ± 34 ^b^
7 dpi	97 ± 17 ^a^	104 ± 17 ^a^	259 ± 76 ^b^	233 ± 47 ^b^
14 dpi	101 ± 16 ^a^	114 ± 28 ^a^	281 ± 99 ^b^	191 ± 51 ^c^
21 dpi	108 ± 23 ^a^	118 ± 29 ^a^	133 ± 32 ^a^	140 ± 30 ^a^

## Data Availability

Data sharing not applicable.

## Sample Availability

Not available.
